# Under the karst: detecting hidden subterranean assemblages using eDNA metabarcoding in the caves of Christmas Island, Australia

**DOI:** 10.1038/s41598-020-78525-6

**Published:** 2020-12-08

**Authors:** Katrina M. West, Zoe T. Richards, Euan S. Harvey, Robert Susac, Alicia Grealy, Michael Bunce

**Affiliations:** 1 Trace and Environmental DNA (TrEnD) Laboratory, School of Molecular and Life Sciences, Curtin University, Bentley, WA 6102 Australia; 2 Western Australian Speleological Group, Nedlands, WA 6909 Australia; 3 Division of Ecology and Evolution, Research School of Biology, The Australian National University, Canberra, ACT 2600 Australia; 4 Environmental Protection Authority, 215 Lambton Quay, Wellington, 6011 New Zealand

**Keywords:** Molecular ecology, Biodiversity

## Abstract

Subterranean ecosystems are understudied and challenging to conventionally survey given the inaccessibility of underground voids and networks. In this study, we conducted a eukaryotic environmental DNA (eDNA) metabarcoding survey across the karst landscape of Christmas Island, (Indian Ocean, Australia) to evaluate the utility of this non-invasive technique to detect subterranean aquatic ‘stygofauna’ assemblages. Three metabarcoding assays targeting the mitochondrial 16S rRNA and nuclear 18S genes were applied to 159 water and sediment samples collected from 23 caves and springs across the island. Taken together, our assays detected a wide diversity of chordates, cnidarians, porifera, arthropods, molluscs, annelids and bryozoans from 71 families across 60 orders. We report a high level of variation between cave and spring subterranean community compositions which are significantly influenced by varying levels of salinity. Additionally, we show that dissolved oxygen and longitudinal gradients significantly affect biotic assemblages within cave communities. Lastly, we combined eDNA-derived community composition and environmental (water quality) data to predict potential underground interconnectivity across Christmas Island. We identified three cave and spring groups that showed a high degree of biotic and abiotic similarity indicating likely local connectivity. This study demonstrates the applicability of eDNA metabarcoding to detect subterranean eukaryotic communities and explore underground interconnectivity.

## Introduction

Subterranean environments are notoriously underexplored. It has been estimated that more than 80% of Australia’s subterranean fauna have yet to be discovered^1^. Subterranean environments can be found above and below the water table. Examples of these environments include caves, cavities, aquifers and anchialine systems. Previous research demonstrates that subterranean environments exhibit a high diversity of (largely invertebrate) taxa that are adapted to a lack of light, and also to variable temperature, nutrient, salinity and dissolved oxygen levels and in some cases, water stratification^2^. Short-range, endemic species are common, as highly fragmented environments pose barriers to gene flow, fostering evolutionary drift over time^3^. Commonly surveyed aquatic ‘stygofauna’, found within aquifers and anchialine systems, include fresh and saltwater fish, eels, gastropods, salamanders, flatworms, beetles, water mites and crustaceans such as amphipods, decapods, isopods, ostracods, copepods and syncarids^4^.

The use of genetic techniques in conjunction with traditional biospeleological sampling and morphological assessment can provide in-depth information in relation to patterns of stygofauna diversity, discrete lineages, colonisation and speciation histories. Biospeleological studies that have incorporated genetic methodology have largely focused on the use of single-source “barcoding” or genome building, where individual specimens are collected and targeted for sequencing^5–10^. However, single-source sequencing remains reliant upon the use of capture-based sampling, which is intrinsically linked to the measurement of biodiversity. Whilst capture-based sampling is fundamental to biospeleological research, it can be hindered in subterranean environments that present difficult to access underground voids and networks. Biospeleological research could therefore benefit from a non-invasive bioassessment tool that complements capture-based sampling in order to gauge stygofauna diversity and distribution.

Environmental DNA (eDNA) metabarcoding has been widely developed within the last few years for use in marine, freshwater and terrestrial environments and has demonstrated its power as an efficient, non-invasive and highly sensitive taxa detection tool^11–14^. However, its application in subterranean environments has not yet been thoroughly explored. Preliminary research has demonstrated the viability of eDNA metabarcoding in detecting multi-species compositions from underground water samples^15–20^, although these have largely focused on microbial communities. Further research is needed to assess the applicability of eDNA metabarcoding for eukaryotic stygofauna detections in subterranean aquatic ecosystems, which through limited surveying and subsequent reference barcoding of biota, may be hindered by incomplete reference databases. There is great potential to expand the use of eDNA metabarcoding, not only for the detection and monitoring of both described and undescribed eukaryotes in subterranean environments, but also to investigate community assemblages across trophic levels and haloclines, the evolution and population diversity of stygofauna, and the interconnectivity of underground ecosystems.

Christmas Island (CI; 10.4475° S, 105.6904° E), one of Australia’s external Indian Ocean territories, forms the pinnacle of an isolated seamount that re-emerged above sea level approximately 5.66–4.49 Ma^21^. A highly developed karst landscape has formed out of imbedded carbonates and approximately 30 accessible caves have been documented, ranging from plateau and freshwater stream caves; to fissure caves, collapsed caves, sea caves, and coastal caves with ocean access points^22^. Rainfall percolates through limestone fractures and solution holes and is largely discharged by coastal and offshore springs, however there are also major inland springs at Waterfall Spring, Ross Hill Gardens and The Dales^22^. Cave fauna are considered to form a significant component of this island’s unique ecosystem^23^ and at present comprise at least 17 out of a total of 253 endemic species documented on CI^24^. However, the diversity and distribution of stygofauna, in particular anchialine fauna, across CI’s extensive underground networks requires further research^25^. This is of particular importance given the use of the karstic landscape for phosphate mining and as a water supply for local households and businesses. The subterranean interconnectivity of the majority of the CI caves are unknown, but it is suspected that caves within close proximity (such as the Whip and Runaway Caves that incidentally also share many species of macrofauna) are connected underground^26^. The need for a comprehensive biodiversity audit of CI’s extensive subterranean habitats makes it an ideal location to conduct a broad eDNA metabarcoding survey of eukaryotic macro-stygofauna. Our main objectives were to: (1) identify putative new occurrence and extend distribution records for CI’s subterranean stygofauna using eDNA metabarcoding; (2) assess variation in community composition of cave and spring sites, and; (3) investigate potential underground interconnectivity across CI by combining biotic and abiotic (environmental parameter) data. Overall, we seek to evaluate the applicability of eDNA metabarcoding as a non-invasive, highly-sensitive tool for biospeleological assessment.

## Methods

### Field sampling

**Figure 1:**
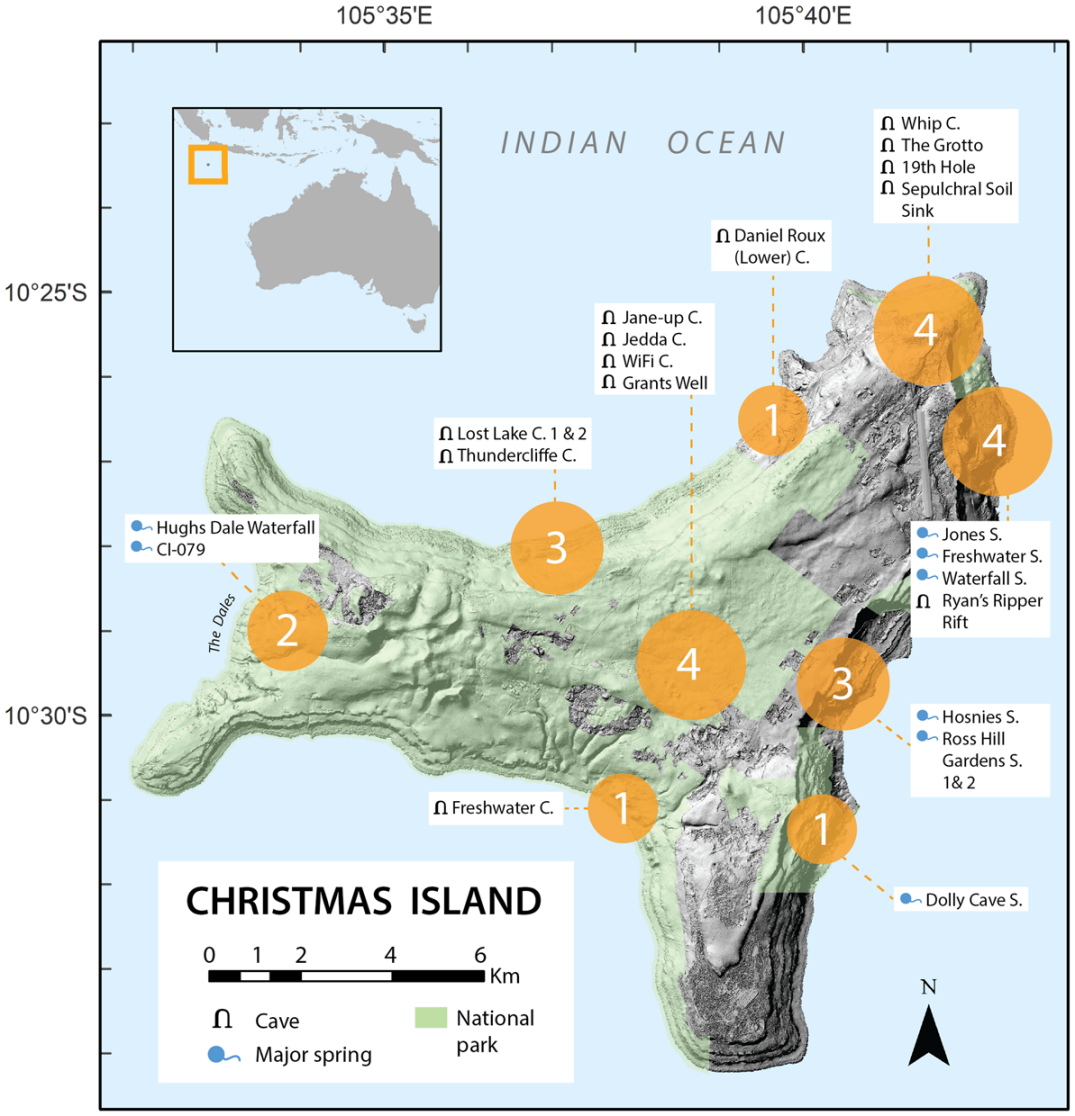
Location of eDNA sampling sites on Christmas Island. Orange spheres give the approximate number and location of sampling sites; topographical symbols indicate whether samples from each respective site were taken from within a cave system or at a surface spring. Hillshade relief and national park boundary data was sourced from Geoscience Australia^53^. Map was produced in ArcGIS Desktop 10.6^54^.

**Figure 2:**
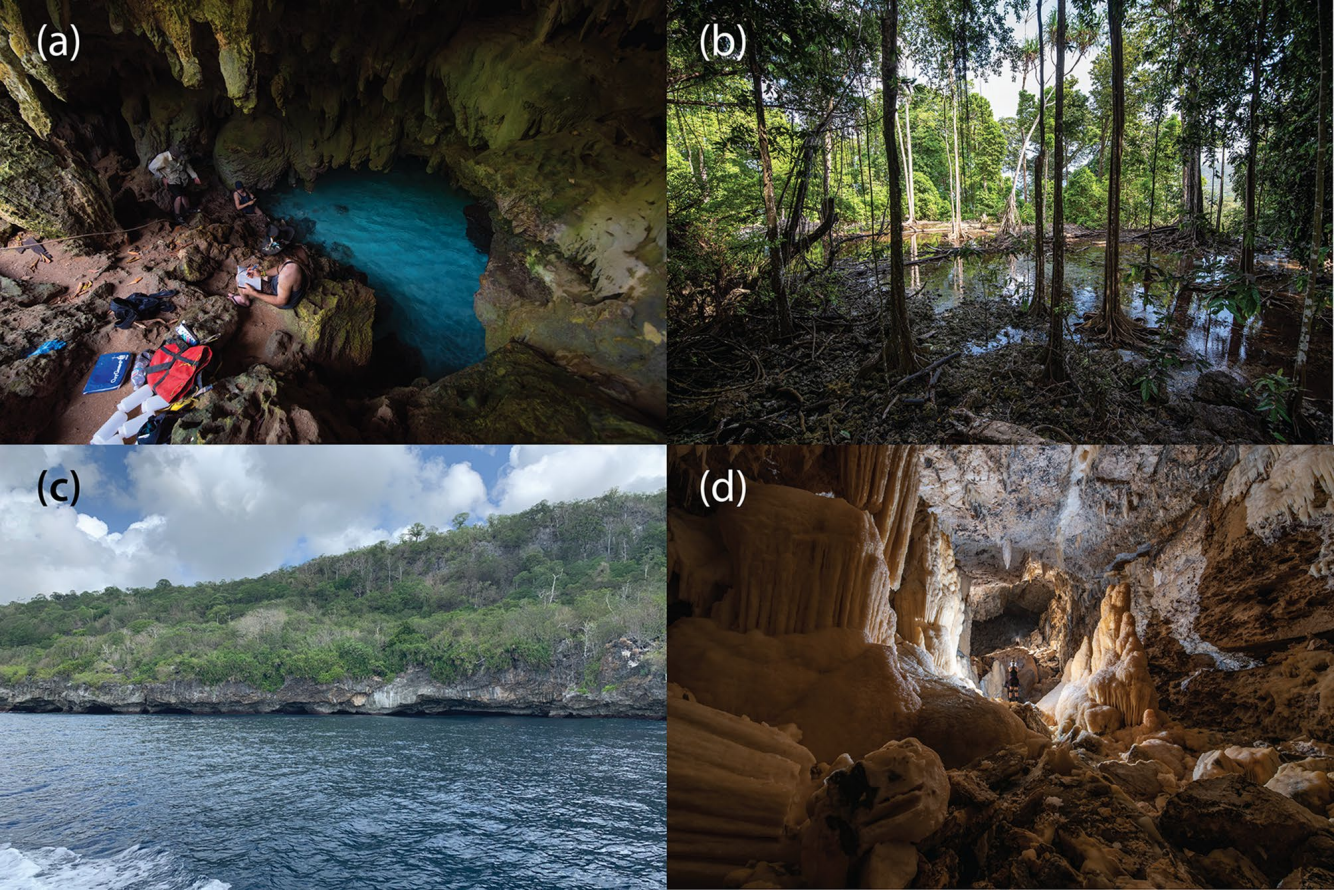
Imagery from eDNA sampling localities across Christmas Island. (**a**) Water and sediment sampling in The Grotto cave site. (**b**) The Dales wetland area where Hugh Dales Waterfall and CI-079 spring sites were sampled. The (**c**) ocean entrance and (**d**) a chamber passage through the extensive Lost Lake Cave system. Photos: Danny Wilkinson and Weidi Koh http://wasg.org.au/.

Six one-litre water replicates and one 50-ml sediment sample were collected from 23 cave and spring sites across CI (Figs. 1 and 2; Table 1) during the late dry season in October 2018, totaling 159 samples across a 110 km^2^ area. Approximately two cave and spring sites were sampled in a day over a two-week period. For public safety and to protect the integrity of the caves, the exact GPS coordinates for our sampled sites are not published here, however they can be requested from Parks Australia (Christmas Island). Sediment could not be taken from the Jedda and Jane-up Cave sites because conduit streams through these plateau caves provide the town water supply and hence access is restricted. However, water samples were taken with permission from the WaterCorp testing taps at these sites. Sediment was sampled at all remaining sites, by collecting a top layer of sediment (approximately 2 cm depth) from underwater sediment sources. Water samples were collected at the surface of each site using bleach sterilised Nalgene bottles and then immediately stored on ice. Each sample was individually filtered across Pall 0.2 μm Supor polyethersulfone membranes using a Pall Sentino Microbiology pump (Pall Corporation, Port Washington, USA), within two hours of collection. A bleach solution (approximately 10% household bleach) was used to clean filtration equipment between samples and a one litre sample of this was filtered at the end of each sampling day to serve as a filtration control throughout laboratory processing. Filter membranes and sediment samples were immediately frozen and stored at −20 °C prior and post-transportation to a quarantine facility within the Trace and Environmental DNA (TrEnD) Laboratory in Perth, Western Australia.

**Table 1:** eDNA site information on Christmas Island.

Site no.	Site name	Type	Brief description	Salinity groups
1	19th Hole	Cave	Anchialine cave with tidal influence. Water table accessed on land via a small cave mouth and down a slope	Mesohaline
2	The Grotto	Cave	Marine coastal cave; strong sea currents and tidal influence. Accessed on land via Golf Course Road and open to the public. Possible connection to Whip Cave^22^	Oligohaline
3	Whip Cave	Cave	Anchialine cave system with tidal influence. Possible connection to The Grotto^22^	Oligohaline
4	Daniel Roux (Lower) Cave	Cave	Anchialine cave system with tidal influence. Water table accessed on land by the lower chamber entrance, down a series of steep rock-mounted steel ladders	Freshwater
5	Freshwater Cave	Cave	Anchialine stream passage cave connecting several chambers	Oligohaline
6	Hugh Dale Waterfall	Spring	Waterfall cascading from tufa spring	Freshwater
7	CI-079 (unnamed)	Spring	Small low cave with spring outflow	Freshwater
8	Sepulchral Soil Sink	Cave	Small entrance cave to deeper chambers with low pools	Mesohaline
9	Waterfall Spring	Spring	Water supply spring south of CI Resort	Freshwater
10	Freshwater Spring	Spring	Spring outflow north of CI Resort	Freshwater
11	Jedda Cave	Cave	Cement steps into doline, pump station for town water supply	Freshwater
12	Jane-up Cave	Cave	Muddy stream passage with high CO_2_; used for town water supply	Freshwater
13	Jones Spring	Spring	Spring with weir, water re-sinks nearby	Freshwater
14	Grants Well	Cave	Well shaft intersecting 20 m below to cave stream passage; high CO_2_	Freshwater
15	Thundercliff Cave	Cave	Sea cave entrance leading to freshwater outflow	Polyhaline
16	Ryan’s Ripper Rift	Cave	Rift cave ending in very deep crystalline edged anchialine water	Oligohaline
17	Hosnies Spring	Spring	Freshwater seepage on shore terrace that feeds a protected mangrove stand	Freshwater
18	Ross Hill Gardens Spring 1	Spring	Captured spring once used for town water supply	Freshwater
19	Ross Hill Gardens Spring 2	Spring	Captured spring once used for town water supply	Freshwater
20	Dolly Cave Spring	Spring	Low muddy cave with spring outflow	Freshwater
21	Lost Lake Cave 1	Cave	Extensive tidal system passages with various large breakdown chambers. Sampled from stream passage approximately 150 m from sea entrance	Oligohaline
22	Lost Lake Cave 2	Cave	Extensive tidal system passages with various large breakdown chambers. Sampled from stream passage far into the cave extent	Oligohaline
23	WiFi Cave	Cave	Deep vertical shaft that is believed to intersect the muddy stream passage between Grants Well and Jane-up Cave	Freshwater

Type refers to whether samples at each respective site were taken from within a cave system or at a surface spring. Salinity classifications for each site were assigned based on salinity readings at the time of sample collection (see Table S1 for full environmental parameter data and CI cave index numbering).

A range of environmental parameters were also taken at the time of sampling from each respective site (Table S1). Measurements of water acidity (pH), temperature (°C), conductivity (mS) and salinity (ppt) were collected using a Hanna HI98129 tester (Hanna Instruments; Victoria, Australia). Air saturation and dissolved oxygen (mg/L) measurements were collected using an OxyGuard Handy Polaris 2 DO Meter (OxyGuard; Farum, Denmark).

### Laboratory processing

DNA was extracted from half of each filter membrane and 250 mg of each sediment sample using a DNeasy PowerLyzer PowerSoil Kit (Qiagen; Venlo, the Netherlands) following the manufacturer’s instructions. This was completed within two weeks post-collection. Remaining half filters and sediment have been stored as an extraction back-up. Filtration controls and extraction blanks, containing no sample, were extracted and processed alongside all samples in order to detect any cross-contamination introduced from the laboratory environment. DNA was amplified using three previously published PCR assays^27–29^ to largely target bony fish, molluscs and arthropods (such as crustaceans and insects) from our mixed environmental samples (see Table 2 for details). Quantitative PCR (qPCR) amplification was performed using fusion tagged primers that consist of an Illumina sequencing adaptor, a unique multiplexing index (8 bp in length) and a primer sequence from each respective assay. All qPCR reactions were prepared in an ultra-clean trace DNA facility and thermocycling was carried out in a physically separated laboratory (see Supplementary Information Sect. 1 for qPCR reagents and conditions).

**Table 2:** Metabarcoding assay information for subterranean eDNA surveys on Christmas Island.

PCR assay	Target taxa	Primer name	Oligonucleotide sequence (5′–3′)	Target length (bp)	Annealing temp (°C)	Primer reference
16S Fish (short)	Bony Fish	16S_FishSyn_Short forward	GACGAGAAGACCCTGTGGAGC	70–140	55	Nester et al.^27^
		16S_FishSyn_Short reverse	CCGYGGTCGCCCCAAC			
16S Crustacean	Crustacean	Crust16S_F (short)	GGGACGATAAGACCCTATA	90–213	51	Berry et al.^28^
		Crust16S_R (short)	ATTACGCTGTTATCCCTAAAG			
18S Universal	Eukaryotes	18S_1F	GCCAGTAGTCATATGCTTGTCT	336–423	52	Pochon et al.^29^
		18S_400R	GCCTGCTGCCTTCCTT			

Three PCR primer sets: 16S Fish (short), 16S Crustacean and 18S Universal corresponding to the mitochondrial 16S rRNA and nuclear 18S regions, were applied to all collected water and sediment samples. In the primer name, “F” refers to the forward primer and “R” to the reverse primer.

Each eDNA sample was amplified in duplicate and pooled into larger amplicon libraries at equimolar ratios based on qPCR ΔRn values. Each library was size-selected (retaining amplicons between 160–450 bp for the 16S assays, and 200–600 bp for the 18S assay) using a Pippin Prep (Sage Science, Beverly, USA), and was then purified using the Qiaquick PCR Purification Kit (Qiagen, Venlo, the Netherlands) following manufacturer instructions. Final libraries were quantified using a Qubit 4.0 Fluorometer (Invitrogen, Carlsbad, USA) and if necessary were diluted to 2 nM prior to sequencing. Libraries were sequenced on either a 300 cycle (for unidirectional sequencing of the 16S amplicons) or 500 cycle (for paired-end sequencing of the 18S amplicons) MiSeq V2 Standard Flow Cell on an Illumina MiSeq platform (Illumina, San Diego, USA), housed in the TrEnD Laboratory at Curtin University, Western Australia.

### Bioinformatics

Unidirectional and unmerged paired-end sequencing reads were demultiplexed using OBITools (v1.2.9)^30^ and the insect package^31^ in RStudio (v1.1.423)^32^, respectively. Demultiplexed data was then quality filtered using the DADA2 pipeline^33^ in RStudio (see Supplementary Information Sect. 2 for bioinformatic parameter details). Resulting amplicon sequence variants (ASV) for each assay were then queried against NCBI’s GenBank nucleotide database^34^ (accessed in 2019) using BLASTn and also against a curated 16S rDNA Western Australian fish database^27^ via Zeus, an SGI cluster, based at the Pawsey Supercomputing Centre in Kensington, Western Australia. Linnaean taxonomic assignments of ASVs were curated using a lowest common ancestor approach (https://github.com/mahsa-mousavi/eDNAFlow/tree/master/LCA_taxonomyAssignment_scripts^35^, see Supplementary Information Sect. 2); consolidated taxa assignments were then additionally categorised based on associated environment and biogeographic distribution data obtained from CI subterranean biodiversity surveys^25,36^ and the World Register of Marine Species (WoRMS)^37^. Putative new occurrence records were additionally assessed for whether all congeneric taxa have been barcoded for the targeted gene region. Any ASVs that were detected in filtration and/or extraction blanks were entirely removed; remaining ASVs that share the exact Linnaean taxonomy assignment were then merged using the phyloseq ‘tax_glom’ function^38^ in RStudio. This produced a taxonomic-based matrix. Read abundance was converted to presence/absence data in PRIMER v7^39^ for subsequent statistical analyses.

### Statistical analyses

Variation in the community composition was firstly tested between the water and sediment samples to determine whether there is a difference in the type and number of taxa detected between the two sample types. The presence-absence data of taxa at each site was converted to a Jaccard similarity matrix and tested for the effect of sample type and site using a two-way crossed PERMANOVA in in the PERMANOVA + add-on^40^ of PRIMER v7. Site variation by sample type was visualised by principal coordinates analysis (PCO) in PRIMER v7. Species accumulation per replicate (of the two sample types) and a comparison of the number of taxa per sample replicate was graphed using the vegan ‘specaccum’ function^41^ and ggplot^42^, respectively, in RStudio. Additionally, a similarity percentage analysis (SIMPER) was conducted in PRIMER v7 to identify contributing taxa to pairwise dissimilarity between the two sample types.

Sampling replicates of both sample types were then merged per site and converted to a Jaccard similarity matrix to examine the overall community composition variation between sites. Taxa accumulation per site was graphed using vegan in RStudio. Distance-based linear model (DistLM) analyses were conducted in the PERMANOVA + add-on using normalised measures of acidity, temperature, salinity and dissolved oxygen, in addition to latitude/longitude, as environmental and spatial predictor variables. These analyses were initially conducted across all site types (cave and spring), and then within cave and spring sites separately. The environmental parameters of conductivity and air saturation were omitted due to collinearity with salinity and dissolved oxygen, respectively. Site variation was visualised by PCO using the stats function ‘cmdscale’ in R Studio; significant predictor variables corresponding to the DistLM analyses were overlaid using the vegan ‘ordisurf’ function^41^. A SIMPER analysis was conducted to identify contributing taxa to pairwise dissimilarity between site type (cave/spring) and salinity. Original salinity readings (ppt) were categorised into the following salinity groups: freshwater ≤ 0.49 ppt, oligohaline 0.5–4.9 ppt, mesohaline 5.0–17.9 ppt, polyhaline 18.0–29.9 ppt, and euhaline (seawater) ≥ 30.0 ppt^43^ (see Table 1 for site salinity groupings). Hierarchical clustering, using group-averaging, and SIMPROF analyses were applied to both the community composition (biotic) and environmental parameter (abiotic; including latitude/longitude) datasets in PRIMER v7, to identify groupings of sites that may potentially reflect underground interconnectivity.

## Results

### Sampling and sequencing statistics

The three metabarcoding assays yielded a total of 35,698,221 sequencing reads across 159 samples. The mean number of filtered sequences (post-quality, denoising and chimera filtering) was 74,014 ± 73,211 per replicate sample for the 16S Fish (short) assay; 66,152 ± 115,417 per replicate sample for the 16S Crustacean assay; and 22,968 ± 16,704 per replicate sample for the 18S Universal assay (Tables S2, S3). The 16S Crustacean assay did yield unbalanced read numbers (post-quality filtering) between some replicates/sites, that given equimolar pooling prior to sequencing, is purported to reflect low template crustacean eDNA at specific subterranean sites (Table S3).

ASVs that were detected in filtration and extraction blanks and/or are common laboratory contaminants were omitted from all samples and subsequent analyses; this included ASVs for minnows (genus: *Phoxinus*), a branching bryozoan (*Fredericella sultana*), human (*Homo sapiens*), junglefowl (*Gallus gallus*), cat (*Felis catus*), pig (*Sus scrofa*), cattle (*Bos taurus*) and turkey (*Meleagris gallopavo*). We also omitted all ASVs for taxa outside of our study scope of subterranean macrofauna; this included any ASV in the domain Bacteria, hairybacks (phylum: Gastrotricha), the kingdom Fungi, ciliates (phylum: Ciliophora), nematodes (phylum: Nematoda), microscopic flatworms (phylum: Platyhelminthes), plants (clade: Streptophyta) and algae (phylum: Cryptophyta).

Taxa accumulation curves based on the addition of each sampling replicate per site (Figure S1) indicated that six 1 L water replicates (chosen a priori to sampling) was not completely sufficient to maximise the observed taxonomic richness at each site. On fitting polynomial curves to the taxa accumulation curves, it was extrapolated that on average 18.6 ± 20.4 one-litre water replicates would be required to maximise observed taxa richness at each site. The addition of a sediment replicate also provided an increment in taxa diversity beyond those detected with water. Therefore, more taxa were likely to be detected if further water and sediment replicates were examined per site. Variation in the composition of taxa detected between the water and sediment sample types was highly significant (*P* = 0.000, *df* = 1; Table S4, Figure S2). A single water sample detected on average a higher number of taxa than a single sediment sample (Figure S3), however, this difference was not statistically significant. A SIMPER analysis of pairwise dissimilarity between the sample types indicated that the water samples were able to detect a large proportion of the overall detected taxa (Table S5), however sediment provided a greater detection rate for yellow nipper crab (*Geograpsus crinipes*) and whiteleg shrimp (*Penaeus vannamei*).

### Overall diversity

**Figure 3:**
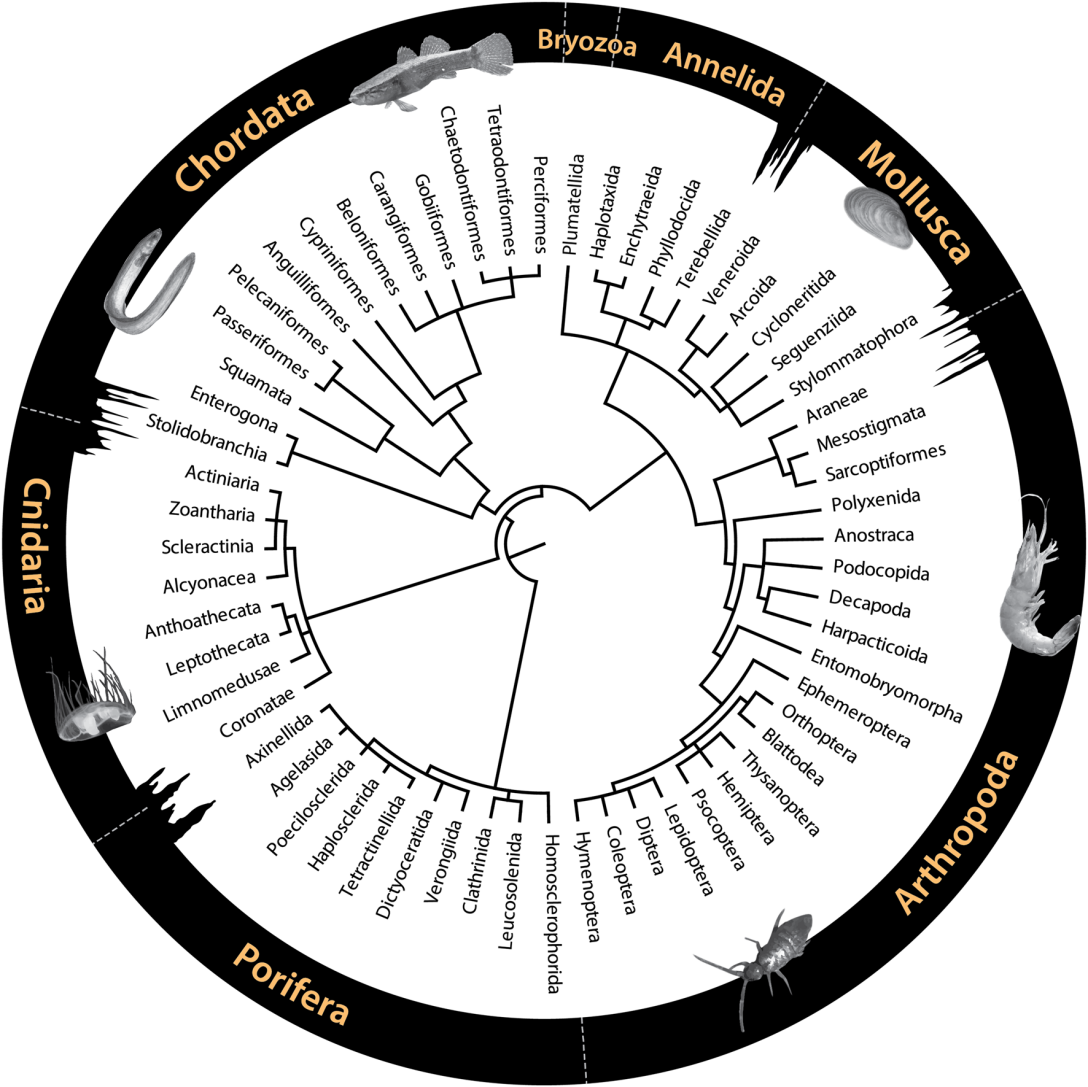
A total ordinal-level dendrogram of chordate, cnidarian, porifera, arthropod, mollusc, annelid and bryozoan taxa detected by multi-assay metabarcoding on 159 eDNA samples collected across Christmas Island.

A total of 25 taxa (1.5 ± 1.0 ASVs per taxa) were detected with the 16S Fish (short) assay, 15 taxa (5.1 ± 7.8 ASVs per taxa) with the 16S Crustacean assay, and 77 taxa (2.0 ± 2.5 ASVs per taxa) with the 18S Universal assay (Table S6). Overall, the three metabarcoding assays yielded 115 identifiable taxa, representing 71 families within 60 orders of the phylums Chordata, Cnidaria, Porifera, Arthropoda, Mollusca, Annelida and Bryozoa (Fig. 3, Table S6). The majority of these taxonomic assignments were resolved to a species level (37.4%), followed by order level (22.6%), genus level (20.8%), family level (17.4%) and class level only (1.7%). The detected taxa were found to be largely associated with marine environments (53.9%), followed by terrestrial (37.4%), freshwater (20.9%) and brackish (14.8%) environments (Table S6). Of these taxa, 64.3% are circumglobal, 6.1% are distributed across the Indo-West Pacific and 5.2% more broadly across the Indo-Pacific, with smaller distributions in Africa, Asia and the eastern Indian Ocean. Taxa accumulation based on the addition of cave/spring sites did not plateau, indicating more taxa are likely to be detected if further sites are examined (Figure S4).

Thirteen bony fish taxa (class: Actinopterygii) were detected (three at family level only, three at genus level only, and seven at a species level) from 11 families within eight orders (Table S6). The assemblage was predominantly comprised of marine fish detected in the anchialine caves; this included snooks (family: Centropomidae), giant trevally (*Caranx ignobilis*), flying fish (family: Exocoetidae), oriental trumpeter whiting (*Sillago aeolus*), black triggerfish (*Melichthys niger*) and halfbeak (*Oxyporhamphus micropterus*). Three notable detections in the freshwater caves were Indonesian shortfin eel (*Anguilla bicolor bicolor*), gudgeon (genus: *Eleotris*) and carp/minnows (family: Cyprinidae); see Table 3 for more information on taxa of conservation, biodiversity and biosecurity importance. We also report three putative new fish occurrence records: Cyprinidae, *Cottus* and *Gobio gobio*. However, the latter species cannot be verified to a species level as not all congeneric taxa for *Gobio* are represented by a reference barcode in the database. Therefore, we cannot rule out that our assignment of *Gobio gobio* may represent a closely-related taxon. See Table S6 for all putative new occurrence records, percent identity and status on whether all congeneric taxa of the respective new occurrence records have been barcoded.

**Table 3:** Taxa of conservation, biodiversity and biosecurity importance.

Common Name	Scientific Name	Importance	Site/s detected	Assay	Associated environment	Biogeographic region
Indonesian shortfin eel	*Anguilla bicolor bicolor*	A single juvenile was previously obtained in Daniel Roux Cave, with larger adults reported by the public in the Dales^36^. Our detection at Dolly Cave Spring in the south-east of CI indicates that this elusive species is more widely distributed across the island	Dolly Cave Spring (20)	16S Fish (short)	Marine, brackish, freshwater	Indo-West Pacific
Gudgeon	*Eleotris*	*Eleotris fusca* has previously been reported in Daniel Roux Cave, Whip Cave, Runaway Cave and Freshwater Cave; these populations exhibit cave adaptations such as a pale body colour, in addition to enlarged pectoral and caudal fins^36,44^. Also occur in caves on many Indo-Pacific Islands^25^	Freshwater Cave (5), Waterfall Spring (9), Freshwater Spring (10), Ryan’s Ripper Rift (16), Hosnies Spring (17)	16S Fish (short)	Freshwater	Circumglobal
Carps, minnows and relatives	Cyprinidae	There were a number of hits for Cyprinidae, however they could not be resolved beyond a family level, exempting the genus *Gobio*. Nonetheless the detection of Cyprinidae is a new occurrence record for CI; this family is not native to Australia and may represent an introduction such as *Cyprinus carpio* in the Murray-Darling River system of eastern Australia. Alternatively, it could represent an unreferenced species in southeast Asia, given the proximity of CI to Indonesia	Hosnies Spring (17), Ross Hill Gardens (18), Dolly Cave Spring (20), Lost Lake Cave (21,22), WiFi Cave (23)	16S Fish (short)	Freshwater	Circumglobal
Freshwater jellyfish	*Craspedacusta sowerbii*	Putative new occurrence record for CI; this freshwater jellyfish is typically found in calm freshwater lakes and reservoirs around the world	Waterfall Spring (9), Dolly Cave Spring (20)	16S Fish (short), 16S Crustacean	Freshwater	Circumglobal
Prawn	*Litopenaeus*	*Litopenaeus vannamei* was detected in this study, however we cannot verify this new occurrence record to a species level, as not all *Litopenaeus* have been barcoded for 18S. Nonetheless, the detection of this genus at CI expands upon previous surveys which give reference to unidentified species of the encompassing family Penaeidae^46^	Whip Cave (3), Lost Lake Cave (21), WiFi Cave (23)	18S Universal	Marine, brackish	Circumglobal
Shrimp	Atyidae	A member of this family, *Antecaridina lauensis*, has previously been identified in Whip Cave, 19th Hole and Runaway Cave^26,36,55,56^. The distribution of this coastal anchialine shrimp may extend to Ryan’s Ripper Rift	Ryan’s Ripper Rift (16)	18S Universal	Freshwater, brackish	Circumglobal
Millipedes	Lophoproctidae	Two species of this family, *Lophoturus speophilus* and *L. humphreysi,* have previously been identified in Jedda Cave and Jane-up Cave, and 19^th^ Hole, respectively^57^. The Lophoproctidae millipedes are commonly found in low light environments such as cave habitats^58^	Jedda Cave (11)	18S Universal	Terrestrial	Circumglobal
Harpacticoid copepod	*Nitokra*	*Nitokra* sp. has previously been identified in Whip Cave and Hendersons Spring^44,56^. The distribution of this copepod now extends to Waterfall Spring. Two species of *Nitokra* have been previously reported in Western Australia; one in the anchialine groundwaters of the Cape Range karst area^59^ and the other in calcrete aquifers broadly across arid WA^60^	Waterfall Spring (9)	18S Universal	Freshwater, brackish	Circumglobal
Slender springtails (collembola)	*Willowsia*	*Willowsia nigromaculata* was detected in this study, however we cannot verify this new occurrence record to a species level, as not all *Willowsia* have been barcoded for 16S. Nonetheless, this genus is a putative new occurrence record for CI; *Willowsia* sp. have previously been recorded in caves on mainland Western Australia^45^. Unidentified species from the encompassing subclass Collembola have previously been identified in Runaway Cave, Jane-up Cave, The Grotto, Grants Well, 19^th^ Hole and Whip Cave^44^	Jane-up Cave (12), Grants Well (14), Thundercliff Cave (15), Ryan’s Ripper Rift (16), Hosnies Spring (17)	16S Crustacean	Terrestrial	Circumglobal

Site number is given in parentheses next to site name.

Forty-seven arthropods (phylum: Arthropoda) were detected (10 at order level only, 11 at family level only, 10 at genus level only and 16 at a species level; Table S6). The assemblage was largely comprised of insects, in addition to arachnids, crustaceans, collembola and millipedes. The majority of the detected arthropods in this study are known to be terrestrial ground-dwelling taxa. We detected a range of land crabs that inhabit the forest floor on CI, but were also spotted in this study within entrances of cave systems; these included the orange-legged crab (*Tuerkayana magnum*), the yellow nipper (*Geograpsus crinipes*), the little nipper (*Geograpsus grayi*) and the purple crab (*Gecarcoidea lalandii*). Of the aquatic taxa, we detected freshwater brine shrimp (*Artemia franciscana*), copepod (*Nitokra*), shrimp (family: Atyidae), ostracods (*Darwinula stevensoni* and *Schlerochilus*) and whiteleg shrimp (*Penaeus vannamei*; see Table 3).

### Community composition and clustering

**Figure 4:**
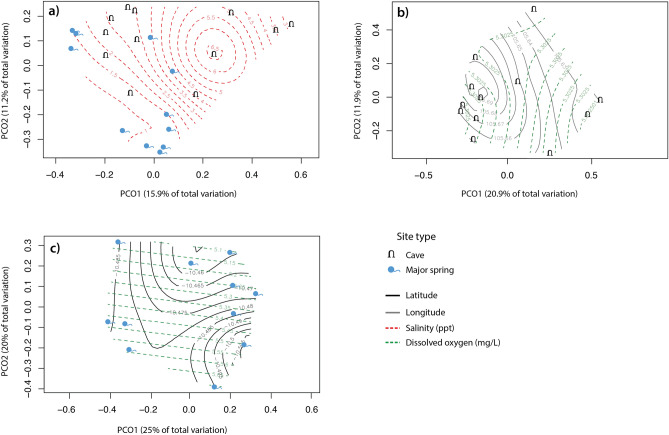
Principal coordinates analysis (PCO) of bony fish composition in (**a**) all sites, (**b**) cave sites only and (**c**) spring sites only. Latitudinal, longitudinal, salinity and dissolved oxygen gradients are overlaid if identified as a predictor variable in corresponding DistLM analyses. The proportion of variation explained by each axis is shown on the axis labels.

**Figure 5:**
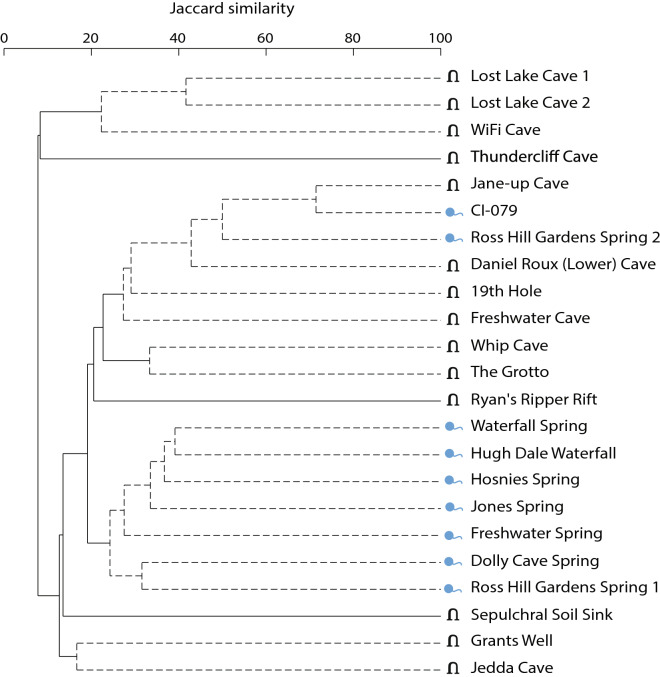
Cluster analysis of CI community composition similarity. Site composition is comprised of all assigned taxa resulting from the three metabarcoding assays. Solid lines indicate groups that the SIMPROF analysis identified were significantly different from each other (*P* < 0.05).

**Figure 6:**
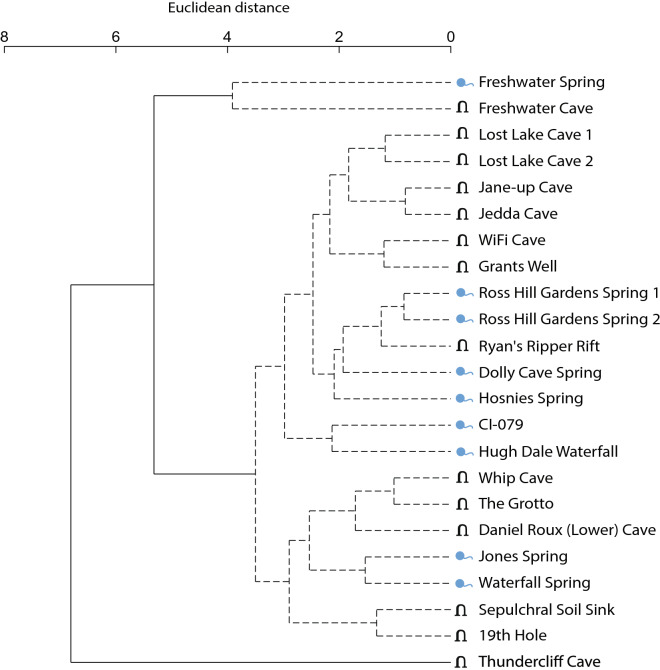
Cluster analysis of CI abiotic environmental similarity. Environmental data incorporated into this analysis included acidity, temperature, salinity, dissolved oxygen, latitude and longitude readings taken from each site at the time of sample collection. Solid lines indicate groups that the SIMPROF analysis identified were significantly different from each other (*P* < 0.05).

A distance-based linear model (DistLM) analysis of community composition across all sites, indicated that site type (cave/spring) explained the highest proportion of fitted variance (9%), followed by salinity (6.1%; Table 4, Table S7). This is visualised in the PCO in Fig. 4a. Taxa richness per site was found not to significantly differ between cave and spring sites (*P* = 0.840, *df* = 1, Table S8, Figure S5), indicating that compositional variation between the site types is not driven by unequal taxa richness. Similarity percentage analysis (SIMPER) was used to identify which subterranean taxa contributed most to pairwise dissimilarity between the site types and between salinity groupings (Tables S9 and S10). In examining cave sites only, we found that compositional dissimilarity is driven by a longitudinal transition, in addition to dissolved oxygen. Cumulatively, these two significant predictor variables explain 21.7% of the total fitted variance between cave assemblages (Fig. 4b, Table 4, Table S7). Within spring sites only, the DistLM identified a latitudinal and dissolved oxygen effect on compositional dissimilarity, however, these were not significant (*P* = 0.103 and *P* = 0.298 respectively, Table 4, Fig. 4c, Table S7). Hierarchical clustering based on the community composition (Jaccard similarity) of all sites revealed a number of discrete groupings; eight were significantly separated by SIMPROF (*P* < 0.05, Fig. 5). For the abiotic environmental data (Euclidean distance), SIMPROF identified three groupings that were significantly different from each other (*P* < 0.05, Fig. 6). A high possibility of local interconnection was attributed to three cave and spring groups which exhibited both biotic and abiotic clustering. These sites were Whip Cave and The Grotto, Jones Spring and Waterfall Spring, and Lost Lake Cave site 1 and site 2.

**Table 4:** Summary table of the distance based linear model (DistLM) analyses for subterranean fauna.

Sites	Predictor	Adj R^2^	Proportion	Cumulative Proportion	*P*
All sites	**Site type (cave/spring)**	0.047	0.090	0.090	0.002**
	**Salinity**	0.066	0.061	0.151	0.041*
	Latitude	0.073	0.048	0.199	0.257
Cave sites only	**Longitude**	0.045	0.119	0.119	0.029*
	**Dissolved oxygen**	0.074	0.098	0.217	0.052*
	Salinity	0.108	0.097	0.314	0.063
	Acidity	0.138	0.089	0.403	0.129
	Temperature	0.176	0.090	0.493	0.127
Spring sites only	Latitude	0.046	0.165	0.165	0.103
	Dissolved oxygen	0.064	0.133	0.298	0.298

These were constructed using a sequential step-wise selection procedure and adjusted R^2^ criterion.

Significant codes are as follows: 0 < 0.001 ‘***’, 0.001 < 0.01 ‘**’, 0.01 < 0.05 ‘*’. The predictor variables highlighted in bold are significant (P < 0.05). Full DistLM results, including marginal tests and best solutions, are provided in Table S7.

## Discussion

### Subterranean diversity

Our multi-assay eDNA metabarcoding approach successfully detected a wide range of chordates, cnidarians, porifera, arthropods, molluscs, annelids and bryozoans from CI’s subterranean habitats. Despite targeting CI’s aquatic stygofauna, our water and sediment samples also produced detection hits for troglofauna (terrestrial cave fauna) that have likely shed DNA into the water below. This demonstrates that water and sediment can be used to detect troglofauna. However, it is likely that other sample types (e.g. soil samples) may provide a greater detection rate for subterranean terrestrial species.

It should also be noted that there was some bycatch of sub-surface terrestrial taxa, such as yellow crazy ant (*Anoplolepis gracilipes*), and ocean-dwelling marine taxa, such as giant trevally (*Caranx ignobilis*) and brown tubular sponge (*Agelas schmidti*). The detection of sub-surface terrestrial taxa was not unsurprising given that rainwater percolates through the karst landscape, potentially carrying organisms or traces of terrestrial DNA into the system. The detection of ocean-dwelling marine taxa may be explained by tidal influences on coastal caves. We retained all taxa in our multivariate site analyses however, as it can be difficult to extricate whether all non-stygobiotic or non-troglobiotic taxa were detected via shared water intrusion through karstic voids or are legitimately part of the subterranean community composition (through potential local adaptations) on CI.

Notable subterranean detections included a putative new occurrence record of Collembola (genus: *Willowsia*) across five sites, which potentially resolves the classification of unidentified Collembola specimens previously collected in Runaway Cave, Jane-up Cave, The Grotto, Grants Well, 19^th^ Hole and Whip Cave^44^, although a specimen of the genus *Cyphoderopsis* has also been identified from an unreferenced cave on the island^45^. Likewise, the detection of prawn (genus: *Litopenaeus*) at three cave sites may also resolve unidentified specimens collected on CI of the encompassing family Penaeidae^46^. An extension in the distribution records of Indonesian shortfin eel (*Anguilla bicolor bicolor*) and gudgeon (genus: *Eleotris*) across CI’s subterranean habitats aids management purposes, particularly as the former is an elusive species that is rarely seen and only reported in two locations^36^, whilst the latter may represent a highly unique lineage exhibiting cave adaptations such as a pale body colour and enlarged pectoral and caudal fins^36,44^.

The overall assemblage also included a total of 21 putative new occurrence records, however their verification requires additional reference barcodes of subterranean fauna in order to rule out incorrect assignments to a closely-related (not yet barcoded) taxon. Subterranean environments, and by extension their fauna, are notoriously under surveyed. As such, only a small number of vouchered specimens have been barcoded for commonly targeted gene regions (i.e. COI, 16S and 12S rRNA). Differences between sexes and life-stages that are classified as different species can also complicate the picture when assembling reference barcode material. One further complication is the high likelihood that vouchered subterranean specimens have been preserved using formalin, such as the two specimens of the rare cave-dwelling flashlight fish (*Photoblepharon palpebratum*) collected from Thundercliff Cave on CI (J. DiBattista, personal communication, January 2019). The preservative unfortunately fragments DNA, modifies bases and creates crosslinks within the DNA^47^, making it challenging to extract and piece together reference sequences. In addition, subterranean environments are highly fragmented habitats, facilitating the evolution of short-range endemic species^3^. These unique lineages require representation in reference databases in order to make robust (species-level) assignments. However, despite an incomplete reference database, this study has demonstrated that eDNA metabarcoding can still reveal a wide diversity of subterranean taxon detections, including some at a species level. Additionally, the detection of unknown species using eDNA (such as the 22.6% of assignments that could not be resolved beyond order level) can be used to direct traditional sampling efforts for specimen acquisition, taxonomic classification and barcoding.

Overall, this eDNA metabarcoding study produced a comparatively high detection rate (115 taxa from 60 orders) to previous CI subterranean surveys that were conducted within a similar sampling time frame^25,36,48^. For example, a submarine and anchialine cave survey using baited traps and visual (SCUBA) surveillance, identified a total of 54 species across 11 coastal CI cave sites, within a 5 week period from 2010 to 2012^36^. A three week survey of CI subterranean environments in 1998 using visual searching, trapping, haul netting and fixed nets, identified 13 aquatic and 17 terrestrial taxa^48^. Lastly, an extensive expedition in 2006 indicated difficulties in obtaining specimens of the ostracod *Humphreysella* and the anchialine shrimp *Procaris*, despite a three-week survey of the original collection site^25^. Environmental DNA metabarcoding may therefore offer a complementary approach to capture-based sampling by detecting elusive subterranean species and providing comprehensive biospeleological assessments.

### Composition and connectivity

The cave and spring systems on CI are distinct and host a different complement of subterranean community assemblages. Given spring water naturally flows through underground and near-surface conduits, we expected spring sites to contain subterranean and potentially surface taxa. Therefore, we anticipated additional taxa in spring sites compared to those detected in the cave sites. However, variation in taxa richness between cave and spring sites was not significant. The spring sites typically produced more sub-surface and terrestrial taxa, whilst the caves sites produced subterranean, largely aquatic, taxa. For example, spring sites were typified by freshwater ostracods (family: Darwinulidae, including *Darwinula stevensoni*), land crabs (*Discoplax magna*, *Gecarcoidea lalandii* and *Geograpsus crinipes*), ticks (order: Sarcoptiformes) and freshwater jellyfish (*Craspedacusta sowerbii*), whilst the cave sites were characterised by slender springtails (*Willowsia*), demosponges (order: Haplosclerida), whiteleg shrimp (*Penaeus vannamei*), and carp/minnows (family: Cyprinidae).

Variation in salinity between the freshwater springs (0.2–0.4ppt) and a combination of freshwater, brackish and saltwater cave sites (0.3–26.0ppt) also had a strong influence on the overall composition. Distinct fauna were found to be associated with each salinity level, for example freshwater and oligohaline sites (0–4.9ppt) were characterised by freshwater ostracods, land crabs, gudgeon (*Eleotris*) and clitellate oligochaete worms (family: Naididae); mesohaline sites (5.0–17.9ppt) by brine shrimp (*Artemia franciscana*), sea sponge (*Iophon*) and giant trevally (*Caranx ignobilis*); and lastly the polyhaline site (18.0–29.9ppt; Thundercliff Cave only) by demosponge (*Callyspongia*), black triggerfish (*Melichthys niger*) and brown tubular sponge (*Agelas schmidti*).

In examining the variation between cave and spring sites separately, the effect of salinity is no longer significant as there is a reduction in salinity variation within each site type. Cave sites were then found to vary on a longitudinal transition across the island—indicating that site dissimilarity increases with distance—in addition to a dissolved oxygen influence. Surface water at most of the cave sites was well oxygenated (ranging between 4.0–5.7 mg/L), although Freshwater Cave (4.0 mg/L) exhibited a lower concentration than previous reports on the island (Humphreys & Eberhard, 2001). Spring sites also varied on a distance-based (albeit latitudinal) and dissolved oxygen transition across the island, although this was not statistically significant. Notably, however, Freshwater Spring exhibited a low dissolved oxygen level of 2.5 mg/L, well below that of any other sampled site. Despite these low oxygen levels which can induce hypoxia in some freshwater fish species^49^, two bony fish taxa (genus: *Eleotris* and family: Leiognathidae) were detected.

Potential underground connectivity was assessed across biotic (community composition) and abiotic (environmental parameter) hierarchical cluster analyses. Sites which exhibit both biotic and abiotic clustering and are presumed to have a high possibility of connection include Whip Cave and The Grotto, Jones Spring and Waterfall Spring, and Lost Lake Cave Site 1 and Site 2. Whip Cave, an anchialine cave, and The Grotto, a coastal marine cave with strong sea currents, are accessed at a distance of 80 m apart. They exhibit highly similar environmental parameters and community composition, and were both noted onsite to be tidally influenced. A possible hydrological connection between Whip Cave and The Grotto has been previously reported^22,44^, in addition to a connection with Runaway Cave^44^. However, we were unable to sample Runaway Cave as part of this project. Our data supports the premise that Whip Cave and The Grotto are connected.

There are no previous reports of connectivity between Jones Spring and Waterfall Spring, however, given they occur in close proximity (1.07 km apart) it is a possibility that they are fed by shared flow systems. Freshwater Spring is situated approximately halfway between Jones and Waterfall Spring and was therefore expected to cluster with these spring sites. Whilst Freshwater Spring is comprised of a similar community composition, it exhibited very different abiotic readings in relation to dissolved oxygen and air saturation. Therefore, we can only suspect based on this evidence that Jones Spring and Waterfall Spring have a high possibility of interconnection. Lost Lake Cave Site 1 and Site 2 were sampled from a continuous stream passage; the former taken approximately 150 m from the sea entrance and the latter sampled far into the cave extent (more than 500 m from sea entrance). It was therefore expected that these two sites would be classified as having a high possibility of interconnection.

Sites with a medium possibility of interconnection (i.e. sites which exhibit either biotic or abiotic clustering) include the plateau sites of Jane-up Cave, Jedda Cave, WiFi Cave and Grants Well, CI-079 and the Hugh Dale Waterfall, and Sepulchral Soil Sink and 19th Hole. Jedda Cave is the mainstay of CI’s water supply and is purported to access a subterranean flow between Grants Well and Jane-up Cave^25,50^. In order to trace the potential flow of water between Grants Well and Jane-up Cave (1.3 km apart), stream water was previously spiked with salt and measured downstream; the through-flow time between the two sites was three hours (400 m/h), confirming their interconnectivity^51^. Abiotic clustering in this study indicates that Jane-up Cave, Jedda Cave, WiFi Cave and Grants Well (located within 1.29 km) are potentially all connected underground, with almost exact water quality readings across the four sites. The spring sites of CI-079 and the Hugh Dale Waterfall, located within The Dales wetland area, also exhibited abiotic clustering and are within a 0.6 km distance of each other. Likewise, the cave sites of Sepulchral Soil Sink and 19^th^ Hole, located east of Flying Fish Cove, exhibited abiotic clustering and are located approximately 1.9 km apart.

The detection of marine taxa can also elucidate sites with ocean connections and therefore potential tidal influence; this included Thundercliff Cave, Lost Lake Cave, The Grotto, 19th Hole, Whip Cave, Sepulchral Soil Sink, Ryan’s Ripper Rift, Hosnies Spring and Ross Hill Gardens Site 1 and 2. All of the cave sites, except for Sepulchral Soil Sink, were noted onsite to have had hourly changes in water level which was attributed to tidal influence. Sepulchral Soil Sink, however, produced salinity readings that were in the range of other tidal influenced sites on CI indicating that it also had an ocean connection. The detection of marine taxa from the three spring sites of Hosnies Spring and Ross Hill Gardens Site 1 and 2 was surprising given they were classified as freshwater based on the surface readings. However, these spring sites are all within 1 km of the coastline; it is therefore possible that they have an ocean connection but exhibit a halocline (salinity) gradient between the groundwater and surface spring.

## Conclusion

The use of eDNA sampling as a bioassessment tool in caves where population dynamics may be extremely fragile to external pressures circumvents the impacts from traditional biospeleological approaches, where specimens must be continually captured for verification beyond initial vouchering and barcoding. Such processes require multiple visits to caves compounding the impacts that are exerted to conservation sensitive areas and fauna. This not only increases levels of impacts exposed to the cave, but increases risk to researchers, and requires additional permits, time and resources to obtain samples. Whilst sampling cave water or sediment for eDNA analyses is logistically easier, karst groundwater is subject to extreme fluctuations in water level during wet and dry seasons/years. This must be taken into consideration when eDNA sampling over successive periods^52^. The power of eDNA metabarcoding lies in its ability to widely amplify a target taxonomic group without specific taxonomic expertise to morphologically classify taxa. This is particularly beneficial in subterranean assessments given the prominence of endemics and taxa that exhibit cave adaptations (e.g. pale body morphs, increased sensory organs, elongated appendages and reduced eyesight).

Subterranean ecosystems are notoriously under surveyed, largely because of logistical difficulties in accessing underground voids and networks. We demonstrated that the application of eDNA metabarcoding assays to water and sediment collected from cave and spring conduits can be used to characterise multi-trophic eukaryotic subterranean diversity from voids that may be inaccessible to conventional biospeleological surveying. We detected a wide range of chordates, cnidarians, porifera, arthropods, molluscs, annelids and bryozoans from freshwater and anchialine spring and cave sites across CI. Community composition was found to vary based on site type (i.e. cave or spring) and salinity; cave sites were additionally influenced by dissolved oxygen and longitudinal gradients. We update distribution information for taxa of biodiversity importance, such as the Indonesian shortfin eel and cave-adapted gudgeon, and potentially resolve unidentified specimen classifications for Collembola and prawn that were previously reported on CI. Lastly, we combined eDNA-derived eukaryotic community composition and environmental (water quality) data to investigate potential underground interconnectivity across CI; based on hierarchical clustering we identified three groups with a high possibility of interconnection. We strongly advocate for ongoing development of subterranean reference databases to facilitate the implementation of eDNA metabarcoding as a biospeleological survey tool, particularly for stygofauna. With this development, we expect that eDNA metabarcoding will be increasingly employed for subterranean multi-trophic surveying, which may reveal food webs and enlighten subterranean ecosystem functioning. We anticipate that this study demonstrates the potential for using multi-marker eDNA metabarcoding approaches for subterranean stygofauna surveying and exploration.

## Supplementary Information

Supplementary Information 1.

Supplementary Information 2.

## Data Availability

Demultiplexed (unfiltered) metabarcoding sequencing data and taxonomic matrix are available for download on Dryad Digital Repository (https://doi.org/10.5061/dryad.d51c5b00s).
