# Supplementary material for: Under the karst: detecting hidden subterranean assemblages using eDNA metabarcoding in the caves of Christmas Island, Australia
Source: Sci Rep. 2020 Dec 8;10:21479. doi: 10.1038/s41598-020-78525-6 (PMC7722930; doi:10.1038/s41598-020-78525-6)
Supplement: Supplementary file 2 — Supplementary Information 2. [file 41598_2020_78525_MOESM2_ESM.pdf]

Supplementary Information

Under the karst: detecting hidden subterranean assemblages using eDNA metabarcoding in the caves of Christmas Island, Australia

Authors: Katrina M. West[1*], Zoe T. Richards[1], Euan S. Harvey[1], Robert Susac[2], Alicia Grealy[3], and Michael Bunce[1,4].

[1] Trace and Environmental DNA (TrEnD) Laboratory, School of Molecular and Life Sciences, Curtin University, Bentley, WA, 6102, Australia.
[2] Western Australian Speleological Group, Nedlands, WA, 6909, Australia.
[3] Division of Ecology and Evolution, Research School of Biology, The Australian National University, ACT, 2600, Australia.
[4] Environmental Protection Authority, 215 Lambton Quay, Wellington 6011, New Zealand.
*Correspondence email: katrina.west@curtin.edu.au

Table S1. Environmental parameter data collected at the time of eDNA sampling.

Site No.	Cave index No.	Site name	Acidity (pH)	Temperature (°C)	Conductivity (mS)	Salinity (ppt)	Air saturation (% sat)	Dissolved Oxygen (mg/L)
1	CI-19	19th Hole	7.9	26.6	14.1	7.1	86.4	5.4
2	CI-1	The Grotto	7.1	26.1	6.4	3.2	87.7	5.7
3	CI-54	Whip Cave	7.0	25.9	5.2	2.6	83.0	5.2
4	CI-3	Daniel Roux (Lower) Cave	7.3	25.7	0.8	0.4	85.5	5.6
5	CI-10	Freshwater Cave	7.2	26.1	1.0	0.5	61.8	4.0
6	-	Hugh Dale Waterfall	7.4	25.2	0.6	0.3	87.3	5.7
7	CI-79	CI-079 (unnamed)	7.5	26.0	0.6	0.3	92.4	5.9
8	CI-103	Sepulchral Soil Sink	7.6	26.3	12.6	6.4	86.0	5.5
9	-	Waterfall Spring	7.5	26.5	0.5	0.2	95.6	6.1
10	-	Freshwater Spring	7.1	26.2	0.8	0.4	39.5	2.5
11	CI-5	Jedda Cave	7.7	26.1	0.5	0.3	80.0	5.1
12	CI-6	Jane-up Cave	7.6	25.8	0.5	0.3	80.4	5.1
13	CI-86	Jones Spring	7.3	26.6	0.7	0.3	86.1	5.5
14	CI-11	Grants Well	7.3	25.1	0.6	0.3	79.5	5.1
15	CI-90	Thundercliff Cave	7.7	26.1	41.6	26.0	79.5	5.0
16	CI-142	Ryan's Ripper Rift	7.7	25.7	3.3	1.6	87.2	5.7
17	CI-75	Hosnies Spring	8.2	25.6	0.6	0.3	87.9	5.7
18	-	Ross Hill Gardens Spring 1	7.5	25.4	0.6	0.3	90.4	5.8
19	-	Ross Hill Gardens Spring 2	7.7	25.6	0.6	0.3	92.5	6.0
20	CI-143	Dolly Cave Spring	7.9	25.8	0.5	0.3	87.5	5.6
21	CI-7	Lost Lake Cave 1	7.4	26.0	9.7	4.8	85.0	5.5
22	CI-7	Lost Lake Cave 2	7.5	26.0	1.2	1.3	86.2	5.6
23	CI-141	WiFi Cave	7.4	25.5	0.6	0.3	82.9	5.3

Supplementary Information Section 1. qPCR reagents and conditions

Quantitative PCR (qPCR) amplification was performed using fusion tagged primers that consist of an Illumina sequencing adaptor, a unique index (8bp in length) and a primer sequence from each respective assay (see Table 2 for assay details). All qPCR reactions were prepared in an ultra-clean trace DNA facility, with each 25 µl reaction containing final concentrations of: 1X AmpliTaq Gold® PCR buffer (Life Technologies, Massachusetts, USA), 2mM $MgCl_2$, 0.25mM dNTPs, 0.2µM each of forward and reverse primers (Integrated DNA Technologies, Australia), 10ug BSA (Fisher Biotec, Australia), 0.6µl of 5X SYBR® Green (Life Technologies), 1U AmpliTaq Gold® DNA Polymerase (Life Technologies), 4µl of eDNA template, and made to volume with Ultrapure™ Distilled Water (Life Technologies). Each qPCR was performed on a StepOnePlus Real-Time PCR System (Applied Biosystems, Massachusetts, USA) under the following conditions: initial denaturation at 95 °C for 5 min, followed by 50 cycles of 30 s at 95 °C, 51-54 °C for 30 s (see respective annealing temperatures in Table 2) and 45 s at 72 °C, with a final extension for 10 min at 72 °C.

Supplementary Information Section 2. Bioinformatic parameters

Unidirectional sequencing reads were demultiplexed using the ngsfilter (allowing up to three mismatches in primer sequences, zero mismatches in unique indexes) and obisplit commands in OBITools (v1.2.9)[1]. Unmerged paired-end sequencing reads were demultiplexed using default parameters in the insect package[2] in RStudio (v1.1.423)[3]. Demultiplexed data was then quality filtered (minimum length=100, maximum expected errors=2, no ambiguous nucleotides), denoised, filtered for chimeras and dereplicated (pool=TRUE) using the DADA2 pipeline[4] in RStudio. The resulting amplicon sequence variant (ASV) fasta file for each assay was then queried against NCBI's GenBank nucleotide database[5] (accessed in 2019) using BLASTn (minimum percentage identity of 90, maximum target sequences of 10, reward value of 1) and also against a curated 16S rDNA Western Australian fish database[6] via Zeus, an SGI cluster, based at the Pawsey Supercomputing Centre in Kensington, Western Australia. Taxonomic assignments of ASVs were curated using a lowest common ancestor (LCA) approach (https://github.com/mahsa-mousavi/eDNAFlow/tree/master/LCA_taxonomyAssignment_scripts)[7]. This collapses assignments to their LCA if the percentage identity between each consecutive hit (set to the top 10 hits) differed by less than one (based on 100% query coverage). We chose this approach as it essentially removes arbitrary percent thresholds for taxonomic assignments. All resulting taxonomic assignments were further classified based on associated environment and biogeographic distribution data obtained from CI subterranean biodiversity surveys[8,9] and the World Register of Marine Species (WoRMS)[10]. Putative new occurrence records were additionally assessed for whether all congeneric taxa have been barcoded for the targeted gene region. Any ASVs that were detected in filtration and/or extraction blanks were entirely removed; remaining ASVs that share the exact taxonomy assignment were then merged using the phyloseq 'tax_glom' function[11] in RStudio. This produced a taxonomic-based matrix; read abundance was converted to presence/absence data in PRIMER v7[12] for subsequent statistical analyses.

Table S2. Mean reads per replicate sample in bioinformatic processing.

Assay	Demultiplexed Input	DADA2 Quality filtered	DADA2 Denoised	Post-chimera removal	Dereplicated unique sequences	ASVs with BLAST Hit
16S Fish (short)	118,973	74,309	74,014	74,014	36	9
16S Crustacean	70,860	66,743	66,159	66,152	15	6
18S Universal	31,833	28,895	27,079	22,968	215	143

Table S3. Total number of quality filtered (post-chimera removal) sequencing reads per replicate sample per assay.

Replicate #	16S Fish (short)	16S Crustacean	18S Universal
1a	130788	12	32630
1b	160508	207	20606
1c	176525	29282	52454
1d	247164	313774	8166
1e	41734	3	29789
1f	207042	13	16791
1g	218477	36	751
2a	3220	6	15783
2b	93110	32341	34151
2c	83565	710158	8981
2d	32266	90840	21342
2e	161445	23	19915
2f	102681	85293	12047
2g	128	3	2657
3a	33751	48	14593
3b	70303	29521	21812
3c	68990	29056	14761
3d	71636	40	40809
3e	125685	120690	18243
3f	70205	185	21595
3g	338944	71	7583
4a	108083	149	81196
4b	26536	6	46514
4c	39095	21	37229
4d	92214	13	20088
4e	114965	36	9438
4f	4477	41	32186
4g	126028	5	61
5a	76568	1	19391
5b	213101	26	31337
5c	155233	81330	31650
5d	215566	94263	47902
5e	150005	513608	11454
5f	140729	2	30123
5g	24	5	39429
6a	44034	46998	60406
6b	29724	4	17687
6c	202716	101693	27472
6d	45611	450499	20166
6e	7904	161640	19154
6f	81566	7	7932
6g	27276	280288	9030
7a	109531	2	20443
7b	66800	50	39990
7c	84510	310461	45932
7d	78200	4	155667

7e	184686	102394	10898
7f	44914	6	16729
7g	390812	5	2593
8a	183034	239	27174
8b	113553	92	15379
8c	135041	95	50161
8d	170442	237	8566
8e	193645	38	13157
8f	115741	292635	22130
8g	22121	35	1
9a	140550	220321	15476
9b	128603	85335	16413
9c	123031	95366	1819
9d	98129	94852	14921
9e	169452	77932	20254
9f	85396	224788	21956
9g	72893	274050	16204
10a	38854	52979	14888
10b	17951	34	14819
10c	35477	130926	29009
10d	7797	19	17822
10e	35936	106134	14519
10f	26659	12	11593
10g	180413	10314	13383
11a	133419	419	19222
11b	51	337970	13794
11c	314	146	22132
11d	81816	517	62426
11e	13816	18	20122
11f	50124	571904	35753
12a	147343	6	9802
12b	673	11	12783
12c	157002	83	11356
12d	3799	7	9939
12e	196512	28229	18867
12f	64882	15	16885
13a	23362	172181	22339
13b	7856	132482	30521
13c	13416	77641	20543
13d	5722	112509	28180
13e	48708	110240	18369
13f	46753	172715	14570
13g	54135	46	13620
14a	86533	264	44134
14b	2020	326	26299
14c	223632	8463	13665
14d	4562	32194	23260
14e	61225	16	5745
14f	89994	5656	13426
14g	64	1	1
15a	121002	136528	20383
15b	59106	2411	28380

15c	101203	132	11568
15d	60786	249	22461
15e	109062	73371	20797
15f	82528	58295	21284
15g	94326	0	19585
16a	103945	39958	19393
16b	55518	41813	24795
16c	2155	310117	26203
16d	69072	238963	20569
16e	90256	57416	19705
16f	137247	56633	22116
16g	3903	16	26824
17a	87169	72366	23475
17b	19360	96055	28125
17c	18869	86437	1
17d	25093	51172	25804
17e	39363	37465	26407
17f	31242	51452	23627
17g	6021	256173	10059
18a	103767	91131	39775
18b	10354	70162	12660
18c	560	40417	22371
18d	18600	78821	18365
18e	36022	98418	27533
18f	2166	94889	18682
18g	72228	59	38484
19a	27	250973	15690
19b	90705	2	24031
19c	37040	77	25991
19d	158470	7	14834
19e	9570	13	16337
19f	74014	5	19835
19g	147958	1	28274
20a	100888	41516	15411
20b	90733	176474	13588
20c	41125	160942	28779
20d	111994	239693	13971
20e	122133	77667	10716
20f	44553	66313	27129
20g	108257	40	19131
21a	65781	1	27889
21b	20968	2	13499
21c	26898	127875	22758
21d	97254	2	38237
21e	138787	0	22435
21f	75	1	28460
21g	259201	0	5467
22a	150718	19872	27832
22b	306422	5	27179
22c	373	2	35185
22d	195556	1	38781
22e	84005	0	54752

22f	1166	43	19086
22g	30	3	1
23a	118601	2	28945
23b	14334	1	59629
23c	19	1	21300
23d	22	1631	17346
23e	123264	1	19639
23f	178373	3	35157
23g	88037	1	20348

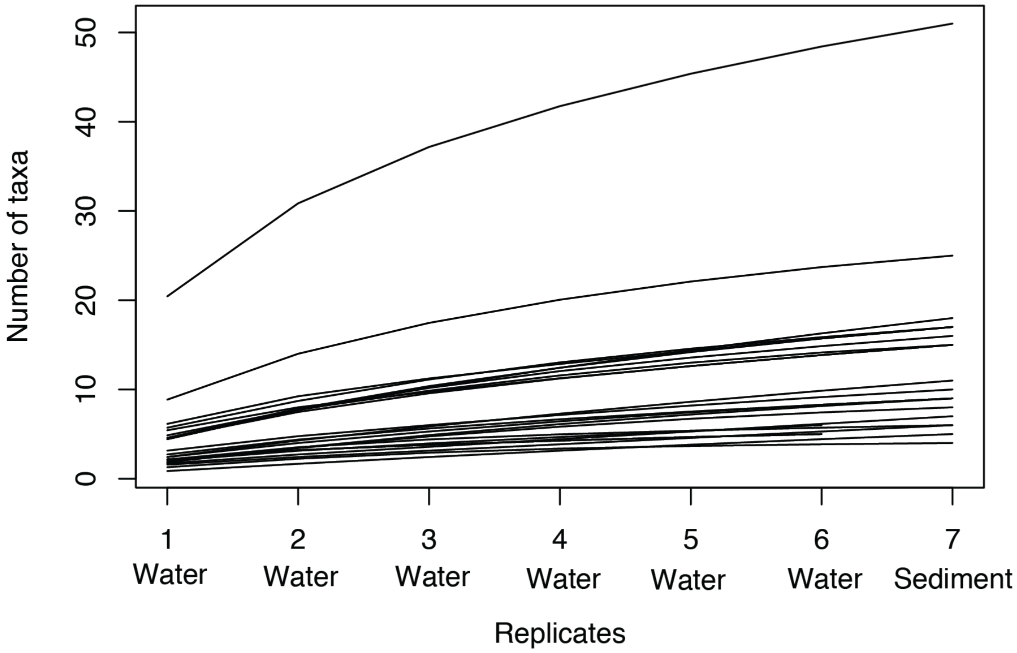

Figure S1. Taxa accumulation curve by the addition of sample replicates (six water and one sediment). Each line represents the addition of replicates at an individual site.

Table S4. Two-way crossed PERMANOVA testing for the effect of site and sample type (water and sediment). Significant *P* value codes are as follows: 0 < 0.001 '***', 0.001 < 0.01 '**', 0.01 < 0.05 '*'.

Source	d.f	Sum of squares	Mean Sq	Pseudo-F	Unique perms	*P*	
Site	22	81952	3725.1	1.3235	9716	**0.0034**	**
Sample type	1	18805	18805	6.6813	9943	**0.0001**	***
Residual	16	45033	2814.6				

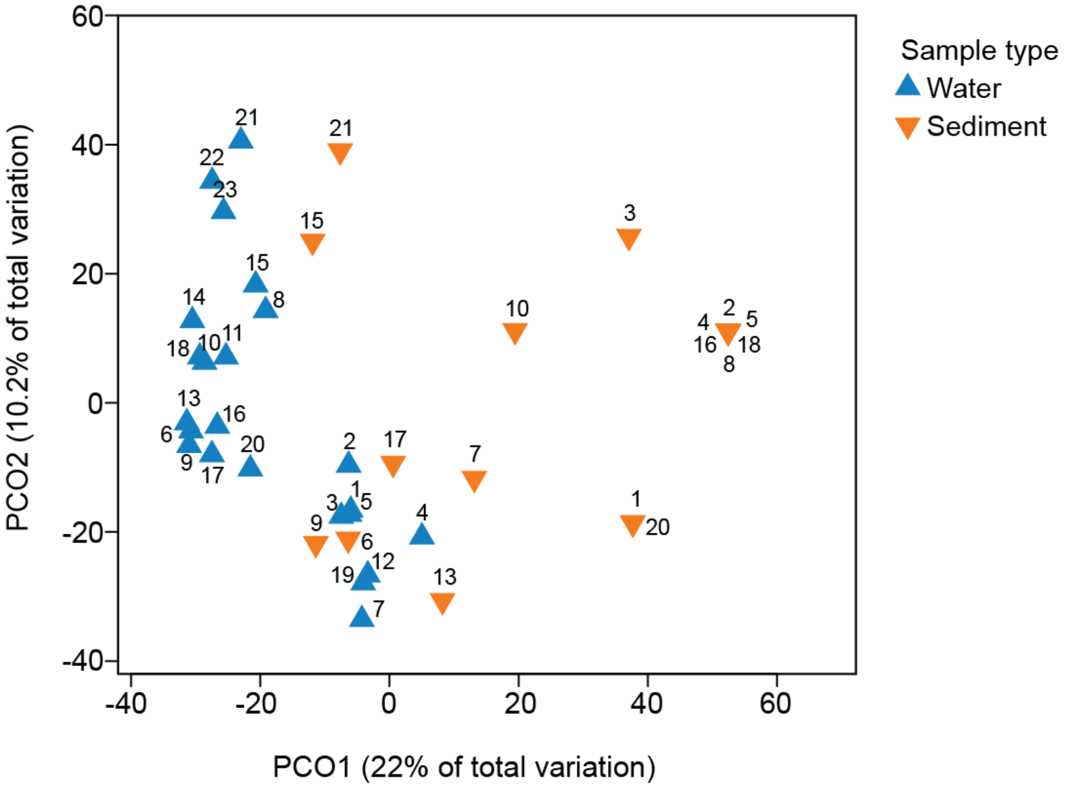

Figure S2. Principal coordinates analysis (PCO) of cave faunal composition by sample type (water and sediment). Sites are numbered next to their respective water (pooled replicates) and sediment samples.

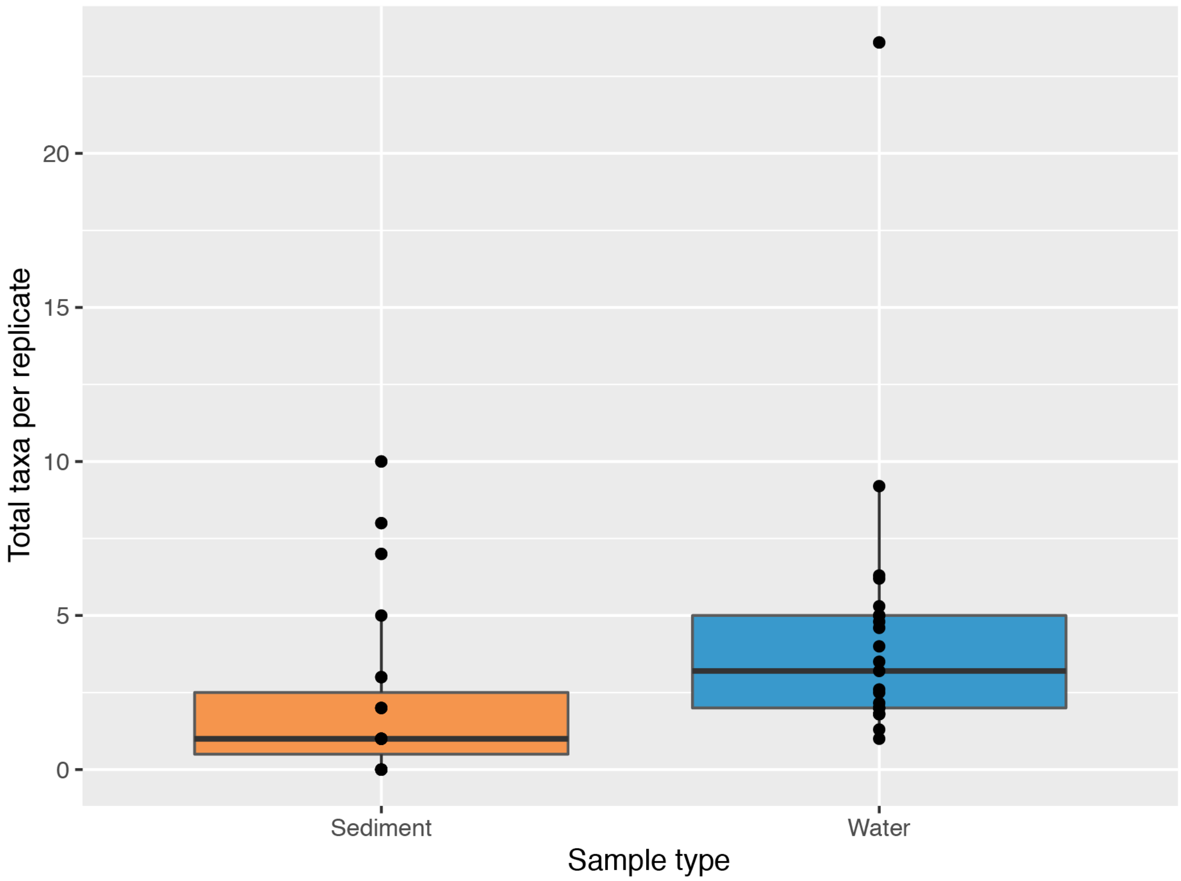

Figure S3. Boxplots of total taxa per sediment and water replicate.

Table S5. Similarity percentage analysis (SIMPER) of CI subterranean taxa. Pairwise dissimilarity between sample types.

Groups	Avg. Diss %	Taxon	Av. Abund in Water	Av. Abund in Sediment	Av. Diss	Diss/ SD	Contrib.%	Cum. %
Water & Sediment	85.85	*Discoplax*	0.96	0.06	7.95	1.68	9.26	9.26
		Gecarcoidea lalandii	0.61	0.29	4.77	0.89	5.55	14.81
		Geograpsus crinipes	0.43	0.88	4.39	0.89	5.11	19.92
		Naididae	0.52	0.18	3.96	0.81	4.62	24.54
		Discoplax magna	0.35	0	3.18	0.64	3.7	28.24
		Darwinulidae	0.3	0.29	2.99	0.76	3.48	31.72
		Sarcoptiformes	0.3	0.24	2.44	0.74	2.85	34.57
		Decapoda	0.26	0.06	2.14	0.58	2.49	37.06
		Anoplolepis gracilipes	0.3	0	1.97	0.54	2.3	39.36
		Gobio gobio	0.26	0	1.85	0.51	2.15	41.52
		Formicidae	0.26	0	1.69	0.56	1.96	43.48
		Psocoptera	0.22	0	1.66	0.5	1.93	45.41
		Shelfordella	0.22	0	1.65	0.44	1.92	47.33
		Penaeus vannamei	0.04	0.12	1.56	0.38	1.81	49.14
		Coptotermes gestroi	0.22	0.06	1.5	0.5	1.75	50.89
		Willowsia nigromaculata	0.22	0	1.41	0.44	1.65	52.54
		Eleotris	0.22	0	1.36	0.49	1.59	54.13
		Exocoetus	0.13	0.06	1.33	0.41	1.55	55.68
		Araneae	0.22	0	1.32	0.51	1.53	57.22
		Demospongiae	0.13	0.06	1.21	0.38	1.41	58.63
		Haplosclerida	0.17	0	1.05	0.4	1.23	59.86
		Cyprinidae	0.13	0	1.05	0.33	1.23	61.08
		Amynthas	0.13	0.06	1.04	0.43	1.21	62.3
		Entomobryomorpha	0.17	0	1.01	0.45	1.18	63.47
		Haplotaxida	0.09	0.06	0.92	0.37	1.07	64.54
		Darwinula stevensoni	0.04	0.12	0.91	0.4	1.06	65.6
		Hemidactylus frenatus	0.13	0	0.86	0.37	1	66.6
		Lepidopsocidae	0.09	0	0.86	0.28	1	67.6
		Lasioseius	0.09	0	0.78	0.28	0.91	68.52
		Craspedacusta sowerbii	0.09	0.06	0.78	0.39	0.91	69.42
		Haliclona cinerea	0.09	0	0.74	0.3	0.86	70.29

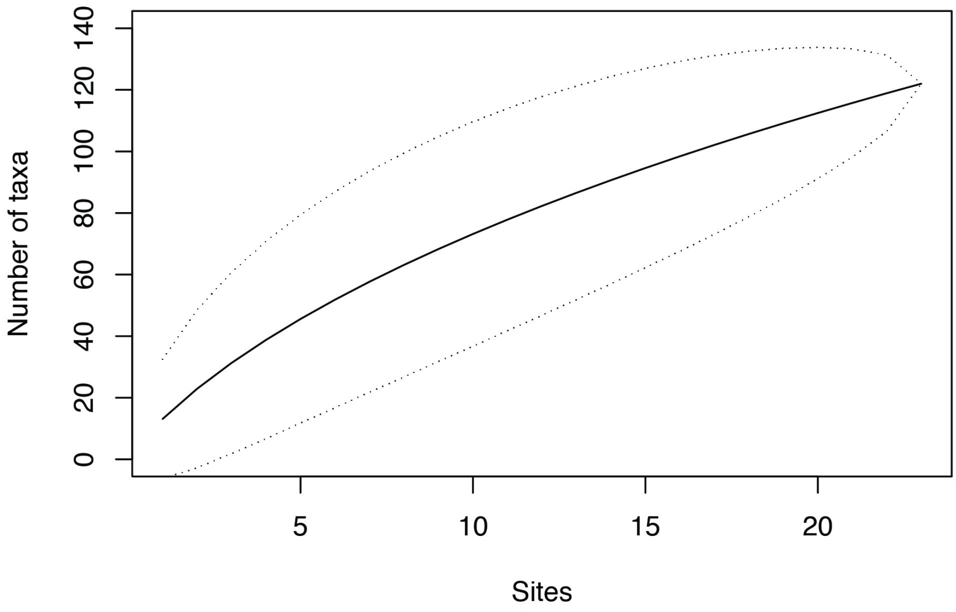

Figure S4. Taxa accumulation curve by the addition of sites (cave/spring). Dotted line represents 95% confidence interval.

Table S7. Distance based linear model (DistLM) analysis of subterranean faunal community variation. These were constructed using a sequential step-wise selection procedure and adjusted R^2 criterion. Significant *P* value codes are as follows: 0 < 0.001 '***', 0.001 < 0.01 '**', 0.01 < 0.05 '*'.

Type of test	Predictor	Adj R^2	Proportion	Cumulative Proportion	*P*	
**All sites**						
Marginal	Latitude		0.051		0.285	
	Longitude		0.042		0.584	
	Acidity		0.048		0.374	
	Temperature		0.049		0.350	
	**Salinity**		0.073		0.015	*
	Dissolved oxygen		0.039		0.724	
	**Site type (cave/spring)**		0.090		0.003	**
Sequential (step-wise)	**Site type (cave/spring)**	0.047	0.090	0.090	0.002	**
	**Salinity**	0.066	0.061	0.151	0.041	*
	Latitude	0.073	0.048	0.199	0.257	
Best solution		**Adj R^2**	**R^2**	**No. Groups**	**Selections**	
		0.073	0.199	3	1, 6, 9	
**Cave sites only**						
Marginal	Latitude		0.084		0.280	
	**Longitude**		0.119		0.030	*
	Acidity		0.083		0.295	
	Temperature		0.090		0.197	
	Salinity		0.100		0.057	
	Dissolved oxygen		0.065		0.776	
Sequential (step-wise)	**Longitude**	0.045	0.119	0.119	0.029	*
	**Dissolved oxygen**	0.074	0.098	0.217	0.053	*
	Salinity	0.108	0.097	0.314	0.063	
	Acidity	0.138	0.089	0.403	0.129	
	Temperature	0.176	0.090	0.493	0.127	
Best solution		**Adj R^2**	**R^2**	**No. Groups**	**Selections**	
		0.176	0.493	5	2-4, 6, 8	
**Spring sites only**						
Marginal	Latitude		0.165		0.113	
	Longitude		0.108		0.717	
	Acidity		0.133		0.377	
	Temperature		0.131		0.397	
	Salinity		0.138		0.317	
	Dissolved oxygen		0.155		0.076	
Sequential (step-wise)	Latitude	0.046	0.165	0.165	0.103	
	Dissolved oxygen	0.064	0.133	0.298	0.311	
Best solution		**Adj R^2**	**R^2**	**No. Groups**	**Selections**	
		0.064	0.298	2	1, 8	

Table S8. One-way ANOVA testing for the effect of site type (cave or spring) on site richness. Significant *P* value codes are as follows: 0 < 0.001 '***', 0.001 < 0.01 '**', 0.01 < 0.05 '*'.

Source	d.f	Sum of squares	Mean Sq	F value	*P*
Site type	1	4.3	4.28	0.042	0.84
Residual	21	2155.5	102.65		

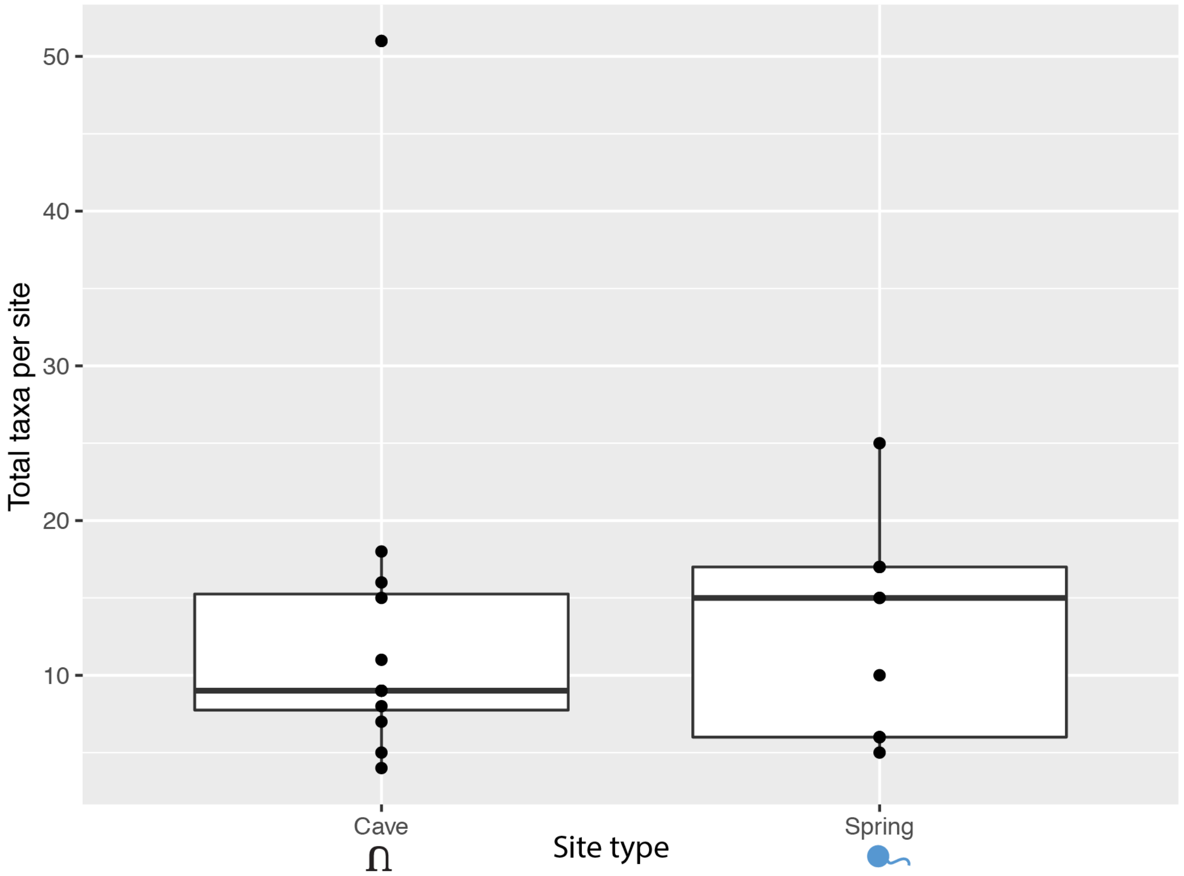

Figure S5. Taxa richness in subterranean fauna across the two site types.

Table S9. Similarity percentage analysis (SIMPER) of CI subterranean taxa. Pairwise dissimilarity between the site types (cave and spring sites).

Groups	Avg. Diss %	Taxon	Av. Abund in Caves	Av. Abund in Springs	Av. Diss	Diss/ SD	Contrib.%	Cum. %
Cave & Spring sites	73.89	Darwinulidae	0	0.89	3.75	1.87	5.08	5.08
		Discoplax magna	0.14	0.67	2.76	1.04	3.73	8.81
		Decapoda	0.07	0.56	2.41	0.93	3.27	12.07
		Sarcoptiformes	0.21	0.67	2.34	1.13	3.17	15.25
		Naididae	0.43	0.67	2.32	0.92	3.13	18.38
		Gecarcoidea lalandii	0.57	0.78	2.1	0.81	2.84	21.22
		Formicidae	0.14	0.44	1.77	0.86	2.4	23.62
		Geograpsus crinipes	0.57	1	1.74	0.72	2.35	25.97
		Gobio gobio	0.21	0.33	1.69	0.74	2.28	28.25
		Exocoetus	0	0.33	1.62	0.62	2.19	30.44
		Anoplolepis gracilipes	0.36	0.22	1.61	0.74	2.18	32.62
		Psocoptera	0.14	0.33	1.59	0.73	2.15	34.77
		Shelfordella	0.21	0.22	1.52	0.61	2.06	36.83
		Coptotermes gestroi	0.21	0.33	1.5	0.75	2.03	38.86
		Araneae	0.14	0.33	1.46	0.75	1.98	40.84
		Eleotris	0.14	0.33	1.43	0.73	1.93	42.77
		Entomobryomorpha	0.07	0.33	1.34	0.72	1.81	44.58
		Willowsia nigromaculata	0.29	0.11	1.24	0.62	1.67	46.25
		Amynthas	0	0.33	1.17	0.67	1.59	47.84
		Darwinula stevensoni	0	0.33	1.17	0.67	1.59	49.43
		Hemidactylus frenatus	0.07	0.22	1.15	0.56	1.56	50.99
		Haplosclerida	0.29	0	0.98	0.56	1.33	52.32
		Penaeus vannamei	0.21	0	0.97	0.48	1.31	53.63
		Cyprinidae	0.21	0	0.89	0.47	1.2	54.83
		Oxyporhamphus micropterus	0	0.22	0.88	0.49	1.2	56.02
		Craspedacusta sowerbii	0	0.22	0.86	0.51	1.16	57.19
		Limnodrilus	0	0.22	0.86	0.51	1.16	58.35
		Araneidae	0.07	0.22	0.83	0.55	1.12	59.47
		Turdus	0	0.22	0.82	0.52	1.12	60.59
		Haplotaxida	0	0.22	0.82	0.52	1.12	61.7
		Lepidopsocidae	0.07	0.11	0.75	0.41	1.02	62.72
		Harpacticoida	0.07	0.11	0.71	0.43	0.96	63.68
		Aphis	0.07	0.11	0.69	0.42	0.94	64.62
		Iophon	0.21	0	0.69	0.46	0.94	65.56
		Demospongiae	0.21	0	0.67	0.46	0.9	66.46
		Haliclona cinerea	0.14	0	0.63	0.39	0.85	67.31
		Lasioseius	0.14	0	0.62	0.38	0.84	68.15
		Polyxenida	0.07	0.11	0.62	0.43	0.84	68.99
		Anthozoa	0.21	0	0.58	0.48	0.79	69.78
		Alcyonacea	0.21	0	0.58	0.48	0.79	70.57

Table S10. Similarity percentage analysis (SIMPER) of CI subterranean taxa. Pairwise dissimilarity between the salinity groups (freshwater, oligohaline, mesohaline & polyhaline).

Groups	Avg. Diss %	Taxon	Av. Abund in Group 1	Av. Abund in Group 2	Av. Diss	Diss/ SD	Contrib. %	Cum. %
Mesohaline & Oligohaline	79.18	*Caranx ignobilis*	0.5	0	2.61	0.92	3.3	3.3
		Atractomorpha	0.5	0	2.61	0.92	3.3	6.6
		Glycyphagidae	0.5	0	2.61	0.92	3.3	9.9
		Stylommatophora	0.5	0	2.61	0.92	3.3	13.2
		Lasioseius	0.5	0.17	2.58	0.92	3.26	16.47
		Gecarcoidea lalandii	0.5	0.67	2.56	0.92	3.23	19.7
		Haplosclerida	0	0.5	2.56	0.94	3.23	22.93
		Iophon	0.5	0.17	2.39	0.93	3.02	25.95
		Artemia franciscana	0.5	0	2.36	0.93	2.98	28.93
Mesohaline & Freshwater	74.77	Naididae	0	0.64	3.38	1.15	4.52	4.52
		Discoplax magna	0	0.57	3.19	1.02	4.27	8.79
		Caranx ignobilis	0.5	0	3.06	0.9	4.09	12.89
		Lasioseius	0.5	0	3.06	0.9	4.09	16.98
		Atractomorpha	0.5	0	3.06	0.9	4.09	21.07
		Glycyphagidae	0.5	0	3.06	0.9	4.09	25.16
		Stylommatophora	0.5	0	3.06	0.9	4.09	29.26
		Gecarcoidea lalandii	0.5	0.64	2.92	0.9	3.9	33.16
		Decapoda	0.5	0.36	2.79	0.91	3.73	36.89
Oligohaline & Freshwater	75.46	*Discoplax magna*	0	0.57	2.54	1.02	3.37	3.37
		Naididae	0.33	0.64	2.47	1	3.27	6.64
		Haplosclerida	0.5	0	2.35	0.92	3.11	9.75
		Darwinulidae	0	0.57	2.16	1.06	2.86	12.61
		Gecarcoidea lalandii	0.67	0.64	2.04	0.83	2.71	15.32
		Sarcoptiformes	0.33	0.43	2.03	0.86	2.69	18.01
		Eleotris	0.33	0.21	1.83	0.74	2.43	20.43
		Geograpsus crinipes	0.67	0.79	1.8	0.75	2.38	22.81
		Anoplolepis gracilipes	0.33	0.29	1.79	0.8	2.37	25.18
Mesohaline & Polyhaline	93.28	*Geograpsus crinipes*	1	0	1.7	41.72	1.82	1.82
		Anoplolepis gracilipes	0	1	1.7	41.72	1.82	3.64
		Willowsia nigromaculata	0	1	1.7	41.72	1.82	5.45
		Coptotermes gestroi	0	1	1.7	41.72	1.82	7.27
		Shelfordella	0	1	1.7	41.72	1.82	9.09
		Melichthys niger	0	1	1.7	41.72	1.82	10.91
		Callyspongia	0	1	1.7	41.72	1.82	12.72
		Fredericella sultana	0	1	1.7	41.72	1.82	14.54
		Agelas schmidti	0	1	1.7	41.72	1.82	16.36
		Oscarella	0	1	1.7	41.72	1.82	18.18
Oligohaline & Polyhaline	82.41	*Melichthys niger*	0	1	1.57	15.66	1.91	1.91
		Fredericella sultana	0	1	1.57	15.66	1.91	3.81
		Agelas schmidti	0	1	1.57	15.66	1.91	5.72
		Oscarella	0	1	1.57	15.66	1.91	7.63
		Hebella contorta	0	1	1.57	15.66	1.91	9.54
		Dynamena crisioides	0	1	1.57	15.66	1.91	11.44
		Scleractinia	0	1	1.57	15.66	1.91	13.35
		Nerillidae	0	1	1.57	15.66	1.91	15.26
		Billardia subrufa	0	1	1.57	15.66	1.91	17.17
Freshwater & Polyhaline	87.89	*Melichthys niger*	0	1	1.62	10.15	1.85	1.85
		Callyspongia	0	1	1.62	10.15	1.85	3.69
		Iophon	0	1	1.62	10.15	1.85	5.54
		Fredericella sultana	0	1	1.62	10.15	1.85	7.38
		Agelas schmidti	0	1	1.62	10.15	1.85	9.23
		Oscarella	0	1	1.62	10.15	1.85	11.08
		Hebella contorta	0	1	1.62	10.15	1.85	12.92
		Haplosclerida	0	1	1.62	10.15	1.85	14.77
		Micronerilla minuta	0	1	1.62	10.15	1.85	16.62

Supplementary references

1. Boyer, F., Mercier, C., Bonin, A., Taberlet, P. & Coissac, E. OBITools: a Unix-inspired software package for DNA metabarcoding. *Mol. Ecol. Resour.* **16**, 176–182 (2014).
2. Wilkinson, S. P., Davy, S. K., Bunce, M. & Stat, M. Taxonomic identification of environmental DNA with informatic sequence classification trees. *PeerJ Prepr.* (2018).
3. R Core Team. RStudio: integrated development for R. *RStudio, Inc., Boston, MA* **42**, (2015).
4. Callahan, B. J. *et al.* DADA2: high-resolution sample inference from Illumina amplicon data. *Nat. Methods* **13**, 581 (2016).
5. Benson, D. A., Karsch-Mizrachi, I., Lipman, D. J., Ostell, J. & Wheeler, D. L. GenBank. *Nucleic Acids Res.* **33**, D34–D38 (2005).
6. Nester, G. M. *et al.* Development and evaluation of fish eDNA metabarcoding assays facilitates the detection of cryptic seahorse taxa (family: Syngnathidae). *Environ. DNA* (2020).
7. Mousavi-Derazmahalleh, M. *et al.* eDNAFlow, an automated, reproducible and scalable workflow for analysis of environmental DNA (eDNA) sequences exploiting Nextflow and Singularity. *Manuscript submitted for publication* (2020).
8. Humphreys, W. F. Subterranean fauna of Christmas Island: habitats and salient features. *Raffles Bull. Zool.* (2014).
9. Hui, T. H., Naruse, T., Fujita, Y. & Kiat, T. S. Observations on the fauna from submarine and associated anchialine caves in Christmas Island, Indian Ocean Territory, Australia. *Raffles Bull. Zool.* (2014).
10. Horton, T. *et al.* World Register of Marine Species (WoRMS). (2018).
11. McMurdie, P. J. & Holmes, S. phyloseq: an R package for reproducible interactive analysis and graphics of microbiome census data. *PLoS One* **8**, e61217 (2013).
12. Clarke, K. R. & Gorley, R. N. *PRIMER v7: User Manual/Tutorial*. (2015).
